# Supplementary material for: Molecular diversity and genetic structure of Saccharum complex accessions
Source: PLoS One. 2020 May 22;15(5):e0233211. doi: 10.1371/journal.pone.0233211 (PMC7244124; doi:10.1371/journal.pone.0233211)
Supplement: S1 Table — (DOCX) [file pone.0233211.s001.docx]

**S1 Table.** **Brazilian Panel of Sugarcane Genotypes (BPSG): accessions, pedigree information, origin and predefined groups of the 254 accessions.**

| ID^1^ | Accession | Female parent^2^ | Male parent^2^ | Origin^2^ | Group^3^ |
| --- | --- | --- | --- | --- | --- |
| 1 | 28NG289 | *S. robustum* | ? | NA | A |
| 2 | 57NG12 | *S. robustum* | ? | NA | A |
| - | 75//09 ERIANTHUS | *Erianthus* spp. | ? | NA | A |
| 3 | AGAUL | *S. sinense* | ? | South Africa | A |
| 4 | AGOULE | *S. barberi* | ? | NA | A |
| 5 | AJA X | *S. officinarum* | ? | NA | A |
| 6 | AKBAR | Co270 | MQ27-1124 | NA | FH |
| 7 | ARCHI | *S. sinense* | ? | Taiwan | A |
| 8 | AROUNDOID B | S. MUTANT | ? | United States | FH |
| 9 | BADILA | S. officinarum | NG9615 | New Guinea | A |
| 10 | BLACK BORNEO | *S. officinarum* | ? | Indonesia | A |
| 11 | CAIANA FITA | *S. officinarum* | ? | NA | A |
| 12 | CAIANA LISTRADA | *S. officinarum* | ? | NA | A |
| 13 | CAIANA RISCADA | *S. officinarum* | ? | NA | A |
| 14 | CAIANA VERDADEIRA | *S. officinarum* | ? | NA | A |
| 15 | CANA ALHO | *S. officinarum* | ? | NA | A |
| 16 | CANA BLANCA | *S. officinarum* | ? | NA | A |
| 17 | CANA MANTEIGA | *S. officinarum* | ? | NA | A |
| 18 | CAYANA | *S. officinarum* | ? | NA | A |
| 19 | CB36-14 | Co213 | ? | Brazil | BB |
| 20 | CB36-24 | POJ2878 | ? | Brazil | BB |
| 21 | CB36-25 | POJ2878 | ? | Brazil | BB |
| 22 | CB36-68 | ? | ? | Brazil | BB |
| 23 | CB40-13 | POJ2878 | Co290 | Brazil | BB |
| 24 | CB40-77 | POJ2878 | Co290 | Brazil | BB |
| 25 | CB41-76 | POJ2878 | ? | Brazil | BB |
| 26 | CB45-155 | Co413 | ? | Brazil | BB |
| 27 | CB45-3 | Co331 | Co290 | Brazil | BB |
| 28 | CB46-47 | POJ2878 | ? | Brazil | BB |
| 29 | CB47-355 | POJ2878 | Co413 | Brazil | BB |
| 30 | CB49-260 | CB44-36 | ? | Brazil | BB |
| 31 | CB53-98 | CB46-40 | ? | Brazil | BB |
| 32 | CERAM RED | *S. officinarum* | ? | NA | A |
| 33 | CHIN | *S. barberi* | ? | India | A |
| 34 | CHINA | *S. sinense* | ? | NA | A |
| 35 | CHUNNE | *S. barberi* | ? | India | A |
| 36 | CINCA77-316 | CP66-346 | CP70-321 | Bolivia | FH |
| 37 | Co285 | Green Sport | *S. spontaneum* | India | FH |
| 38 | Co290 | Co221 | D74 | India | FH |
| 39 | Co331 | Co213 | Co214 | India | FH |
| 40 | Co419 | POJ2878 | Co290 | India | FH |
| 41 | Co449 | Co331 | POJ2878 | India | FH |

**S1 Table.** Continued.

| ID^1^ | Accession | Female parent^2^ | Male parent^2^ | Origin^2^ | Group^3^ |
| --- | --- | --- | --- | --- | --- |
| 42 | Co740 | P3247 | P4775 | India | FH |
| 43 | Co997 | Co683 | P63-32 | India | FH |
| 44 | CP51-22 | F36-819 | CP33-372 | United States | FH |
| 45 | CP52-68 | CP29-320 | CP38-34 | United States | FH |
| 46 | CP53-76 | F36-819 | CP36-46 | United States | FH |
| 47 | CR72106 | EROS | B49-119 | Dominican Republic | FH |
| 48 | CREOULA | *S. officinarum* | ? | NA | A |
| 49 | CRIOLLA RAYADA | *S. officinarum* | ? | NA | A |
| 50 | CRISTALINA | *S. officinarum* | ? | NA | A |
| 51 | D11/35 | *S. officinarum* | ? | United States | A |
| 52 | D152 | B. cheribon | Batjan | NA | FH |
| 53 | D625 | Dyer | ? | NA | FH |
| 54 | EK28 | POJ100 | EK2 | Java | FH |
| 55 | F150 | PT43-52 | Nco-310 | Taiwan | FH |
| 56 | F31-962 | Co281 | CP27-108 | Taiwan | FH |
| 57 | F36-819 | F31-962 | POJ2878 | Taiwan | FH |
| 58 | F76-1762 | *Miscanthus* spp. | *S. officinarum* | NA | A |
| 59 | FORMOSA | *S. officinarum* | ? | Taiwan | A |
| 60 | GANDACHENI | *S. barberi* | ? | India | A |
| 61 | GREEN GERMAN | *S. officinarum* | ? | NA | A |
| - | H. KAWANDANG | *Erianthus* spp. | ? | Indonesia | A |
| 62 | H53-3989 | H48-3717 | ? | United States | FH |
| 63 | H59-1966 | H50-676 | H49-3646 | United States | FH |
| 64 | HJ5741 | H40-1184 | ? | United States | FH |
| 65 | IAC48-65 | CP27-108 | ? | Brazil | BB |
| 66 | IAC49-131 | CP27-108 | ? | Brazil | BB |
| 67 | IAC51-205 | POJ2878 | ? | Brazil | BB |
| 68 | IAC52-150 | Co419 | Co285 | Brazil | BB |
| 69 | IAC58-480 | POJ2878 | CP44-101 | Brazil | BB |
| 70 | IAC64-257 | Co419 | IAC49-131 | Brazil | BB |
| 71 | IAC68-12 | Co419 | IAC52-179 | Brazil | BB |
| 72 | IAC82-2045 | IAC65-113 | IAC52-150 | Brazil | BB |
| 73 | IAC82-3092 | CB41-76 | IAC68-12 | Brazil | BB |
| 74 | IAC83-4157 | IAC68-12 | SP70-1143 | Brazil | BB |
| 75 | IAC86-2210 | CP52-58 | Co798 | Brazil | BB |
| 76 | IAC87-3396 | Co740 | SP70-1143 | Brazil | BB |
| 77 | IAC91-1099 | RB785148 | ? | Brazil | BB |
| 78 | IS76-155 | *S. officinarum* | ? | NA | A |
| 79 | IJ76-360 | *S. edule* | ? | NA | A |
| 80 | IJ76-293 | *S. robustum* | ? | Indonesia | A |
| 81 | IJ76-313 | *S. officinarum* | ? | Indonesia | A |
| 82 | IJ76-314 | *S. robustum* | *S. officinarum* | Indonesia | A |
| 83 | IJ76-317 | *S. officinarum* | ? | Indonesia | A |

**S1 Table.** Continued.

| ID^1^ | Accession | Female parent^2^ | Male parent^2^ | Origin^2^ | Group^3^ |
| --- | --- | --- | --- | --- | --- |
| 84 | IJ76-318 | *S. robustum* | ? | Indonesia | A |
| 85 | IJ76-325 | *S. officinarum* | ? | Indonesia | A |
| 86 | IJ76-326 | *S. officinarum* | ? | Indonesia | A |
| 87 | IJ76-418 RED | *S. officinarum* | ? | Indonesia | A |
| 88 | IJ76-560 | *S. officinarum* | ? | Indonesia | A |
| - | IM76-227 | *Erianthus* spp. | ? | Indonesia | A |
| 89 | IM76-228 | *S. robustum* | ? | Indonesia | A |
| 90 | IM76-229 | *S. robustum* | ? | Indonesia | A |
| 91 | IN84-103 | *S. officinarum* | ? | Indonesia | A |
| 92 | IN84-104 | *S. robustum* | ? | Indonesia | A |
| 93 | IN84-105 | *S. officinarum* | ? | Indonesia | A |
| 94 | IN84-106 | *S. officinarum* | ? | Indonesia | A |
| 95 | IN84-117 | *S. robustum* | ? | Indonesia | A |
| 96 | IN84-46 | *S. officinarum* | ? | Indonesia | A |
| 97 | IN84-58 | *S. spontaneum* | ? | Indonesia | A |
| - | IN84-73 | *Erianthus* spp. | ? | NA | A |
| - | IN84-77 | *Erianthus* spp. | ? | NA | A |
| 98 | IN84-82 | *S. spontaneum* | ? | Indonesia | A |
| - | IN84-83 | *Erianthus* spp. | ? | NA | A |
| 99 | IN84-88 | *S. spontaneum* | ? | Indonesia | A |
| 100 | KAVANGIRA | *S. sinense* | ? | NA | A |
| 101 | KRAKATAU | *S. spontaneum* | ? | NA | A |
| 102 | L60-14 | CP52-1 | CP48-103 | United States | FH |
| 103 | LAUKONA | *S. officinarum* | ? | United States | A |
| 104 | LOUSER | *S. officinarum* | ? | NA | A |
| 105 | MALI | 405N1133 | 33MQ371 | NA | FH |
| 106 | MANAII | *S. officinarum* | ? | NA | A |
| 107 | MANERIA | *S. sinense* | ? | India | A |
| 108 | MUNTOK JAVA | *S. officinarum* | ? | Indonesia | A |
| 109 | MZ-151 | *S. officinarum* | ? | NA | A |
| 110 | NA56-79 | Co419 | Co419 | Argentina | FH |
| 111 | Nco-310 | Co421 | Co312 | South Africa | FH |
| 112 | NG21-17 | *S. officinarum* | ? | New Guinea | A |
| 113 | NG21-21 | *S. officinarum* | ? | New Guinea | A |
| 114 | NG57-221 | *S. officinarum* | ? | New Guinea | A |
| 115 | NG77-18 | *S. officinarum* | ? | New Guinea | A |
| 116 | POJ161 | Black Cheribon | Chunnee | Indonesia | FH |
| 117 | POJ2878 | POJ2364 | EK28 | Indonesia | FH |
| 118 | Q117 | 58N829 | Q77 | Australia | FH |
| 119 | Q165 | Q 117 | CP 33/372 | Australia | FH |
| 120 | Q70 | POJ2878 | H31-2484 | Australia | FH |
| 121 | R570 | R445 | H32-8560 | Reunion Island | FH |
| 122 | RAGNAR | *S. officinarum* | *S. spontaneum* | United States | FH |

**S1 Table.** Continued.

| ID^1^ | Accession | Female parent^2^ | Male parent^2^ | Origin^2^ | Group^3^ |
| --- | --- | --- | --- | --- | --- |
| 123 | RB002601 | RB75126 | Q107 | Brazil | BB |
| 124 | RB002700 | SP80-1816 | ? | Brazil | BB |
| 125 | RB002754 | RB835205 | ? | Brazil | BB |
| 126 | RB721012 | Co331 | ? | Brazil | BB |
| 127 | RB72199 | NCo334 | ? | Brazil | BB |
| 128 | RB72454 | CP53-76 | ? | Brazil | BB |
| 129 | RB725053 | Co775 | ? | Brazil | BB |
| 130 | RB725828 | NA56-79 | ? | Brazil | BB |
| 131 | RB732577 | Nco-376 | ? | Brazil | BB |
| 132 | RB735200 | Co331 | ? | Brazil | BB |
| 133 | RB735220 | CB61-99 | ? | Brazil | BB |
| 134 | RB735275 | IAC49-131 | ? | Brazil | BB |
| 135 | RB736018 | M253/48 | ? | Brazil | BB |
| 136 | RB739359 | IANE55-34 | ? | Brazil | BB |
| 137 | RB739735 | CB52-179 | ? | Brazil | BB |
| 138 | RB75126 | C278 | ? | Brazil | BB |
| 139 | RB765418 | M253/48 | ? | Brazil | BB |
| 140 | RB785148 | IAC47-31 | ? | Brazil | BB |
| 141 | RB785750 | TUC67-11 | ? | Brazil | BB |
| 142 | RB805276 | NA56-79 | ? | Brazil | BB |
| 143 | RB806043 | NA56-79 | ? | Brazil | BB |
| 144 | RB815521 | NA56-79 | ? | Brazil | BB |
| 145 | RB815627 | IAC49-131 | NA56-79 | Brazil | BB |
| 146 | RB815690 | IAC49-131 | NA56-79 | Brazil | BB |
| 147 | RB825317 | L60-14 | CB47-355 | Brazil | BB |
| 148 | RB825336 | H53-3989 | ? | Brazil | BB |
| 149 | RB825548 | F150 | ? | Brazil | BB |
| 150 | RB83100 | NA56-79 | SP70-1143 | Brazil | BB |
| 151 | RB83102 | NA56-79 | SP70-1143 | Brazil | BB |
| 152 | RB83160 | NA56-79 | SP70-1143 | Brazil | BB |
| 153 | RB835019 | RB72454 | NA56-79 | Brazil | BB |
| 154 | RB835054 | RB72454 | NA56-79 | Brazil | BB |
| 155 | RB835089 | RB72454 | NA56-79 | Brazil | BB |
| 156 | RB835205 | Co740 | ? | Brazil | BB |
| 157 | RB835486 | L60-14 | ? | Brazil | BB |
| 158 | RB845197 | RB72454 | SP70-1143 | Brazil | BB |
| 159 | RB845210 | RB72454 | SP70-1143 | Brazil | BB |
| 160 | RB845239 | RB72454 | SP70-1143 | Brazil | BB |
| 161 | RB845257 | RB72454 | SP70-1143 | Brazil | BB |
| 162 | RB845286 | RB72454 | SP70-1143 | Brazil | BB |
| 163 | RB855002 | SP70-1143 | RB72454 | Brazil | BB |
| 164 | RB855035 | L60-14 | SP70-1284 | Brazil | BB |
| 165 | RB855036 | RB72454 | SP70-1143 | Brazil | BB |

**S1 Table.** Continued.

| ID^1^ | Accession | Female parent^2^ | Male parent^2^ | Origin^2^ | Group^3^ |
| --- | --- | --- | --- | --- | --- |
| 166 | RB855063 | TUC71-7 | SP70-1143 | Brazil | BB |
| 167 | RB855070 | SP70-1143 | TUC71-7 | Brazil | BB |
| 168 | RB855077 | SP70-1143 | TUC71-7 | Brazil | BB |
| 169 | RB855113 | SP70-1143 | RB72454 | Brazil | BB |
| 170 | RB855156 | RB72454 | TUC71-7 | Brazil | BB |
| 171 | RB855196 | RB72454 | TUC71-7 | Brazil | BB |
| 172 | RB855206 | RB72454 | TUC71-7 | Brazil | BB |
| 173 | RB855350 | RB72454 | ? | Brazil | BB |
| 174 | RB855357 | RB72454 | ? | Brazil | BB |
| 175 | RB855453 | TUC71-7 | ? | Brazil | BB |
| 176 | RB855463 | RB72454 | ? | Brazil | BB |
| 177 | RB855465 | RB72454 | ? | Brazil | BB |
| 178 | RB855511 | SP71-1406 | ? | Brazil | BB |
| 179 | RB855533 | NA56-79 | ? | Brazil | BB |
| 180 | RB855536 | SP70-1143 | RB72454 | Brazil | BB |
| 181 | RB855546 | SP70-1143 | RB72454 | Brazil | BB |
| 182 | RB855563 | TUC71-7 | SP70-1143 | Brazil | BB |
| 183 | RB855574 | SP70-1143 | TUC71-7 | Brazil | BB |
| 184 | RB855589 | SP70-1143 | TUC71-7 | Brazil | BB |
| 185 | RB855595 | SP70-1143 | TUC71-7 | Brazil | BB |
| 186 | RB855598 | SP70-1143 | TUC71-7 | Brazil | BB |
| 187 | RB865214 | RB735220 | SP71-6163 | Brazil | BB |
| 188 | RB867515 | RB72454 | ? | Brazil | BB |
| 189 | RB925268 | RB855511 | ? | Brazil | BB |
| 190 | RB925345 | H59-1966 | ? | Brazil | BB |
| 191 | RB92579 | RB75126 | RB72199 | Brazil | BB |
| 192 | RB935744 | RB835089 | RB765418 | Brazil | BB |
| 193 | RB965902 | RB855536 | RB855453 | Brazil | BB |
| 194 | RB965917 | RB855453 | RB855536 | Brazil | BB |
| 195 | RB966928 | RB855156 | RB815690 | Brazil | BB |
| 196 | RB975148 | RB815521 | RB835486 | Brazil | BB |
| 197 | RB975157 | RB855563 | RB735200 | Brazil | BB |
| 198 | RB975184 | RB72454 | SP79-1011 | Brazil | BB |
| 199 | RB975201 | RB855113 | ? | Brazil | BB |
| 200 | RB975932 | SP80-185 | RB855206 | Brazil | BB |
| 201 | RB975952 | RB835486 | RB825548 | Brazil | BB |
| 202 | RB985476 | H53-3989 | RB855206 | Brazil | BB |
| 203 | SABURA | *S. officinarum* | ? | NA | A |
| 204 | SAC OFFIC 8272 | *S. officinarum* | ? | Malasia | A |
| 205 | SAC OFFIC 8276 | *S. officinarum* | ? | Malasia | A |
| 206 | SAC OFFIC 8280 | *S. officinarum* | ? | Malasia | A |
| 207 | SAC OFFIC 8284 | *S. officinarum* | ? | Malasia | A |
| 208 | SES205A | *S. spontaneum* | ? | India | A |

**S1 Table.** Continued.

| ID^1^ | Accession | Female parent^2^ | Male parent^2^ | Origin^2^ | Group^3^ |
| --- | --- | --- | --- | --- | --- |
| 209 | SP70-1005 | IAC48-65 | ? | Brazil | BB |
| 210 | SP70-1078 | IAC48-65 | ? | Brazil | BB |
| 211 | SP70-1143 | IAC48-65 | ? | Brazil | BB |
| 212 | SP70-1284 | CB41-76 | ? | Brazil | BB |
| 213 | SP70-1423 | CB41-76 | ? | Brazil | BB |
| 214 | SP70-3370 | CP53-17 | ? | Brazil | BB |
| 215 | SP71-1406 | NA56-79 | ? | Brazil | BB |
| 216 | SP71-6163 | NA56-79 | ? | Brazil | BB |
| 217 | SP71-6949 | NA56-79 | ? | Brazil | BB |
| 218 | SP71-799 | NA56-79 | ? | Brazil | BB |
| 219 | SP72-4928 | CP52-48 | ? | Brazil | BB |
| 220 | SP77-5181 | HJ57-41 | ? | Brazil | BB |
| 221 | SP79-1011 | NA56-79 | Co775 | Brazil | BB |
| 222 | SP79-2233 | H56-2954 | ? | Brazil | BB |
| 223 | SP79-2312 | SP71-6106 | ? | Brazil | BB |
| 224 | SP79-2313 | SP71-6106 | ? | Brazil | BB |
| 225 | SP79-6192 | SP70-3518 | ? | Brazil | BB |
| 226 | SP80-1520 | H48-3166 | SP71-1088 | Brazil | BB |
| 227 | SP80-180 | B3337 | ? | Brazil | BB |
| 228 | SP80-1816 | SP71-1088 | H57-5028 | Brazil | BB |
| 229 | SP80-1836 | SP71-1088 | H57-5028 | Brazil | BB |
| 230 | SP80-1842 | SP71-1088 | H57-5028 | Brazil | BB |
| 231 | SP80-185 | BO17 | ? | Brazil | BB |
| 232 | SP80-3280 | SP71-1088 | H57-5028 | Brazil | BB |
| 233 | SP81-1763 | co775 | NA56-79 | Brazil | BB |
| 234 | SP81-3250 | CP70-1547 | SP71-1279 | Brazil | BB |
| 235 | SP83-2847 | HJ5741 | SP70-1143 | Brazil | BB |
| 236 | SP83-5073 | SP71-1406 | SP71-1088 | Brazil | BB |
| 237 | SP86-155 | SP78-3081 | ? | Brazil | BB |
| 238 | SP89-1115 | CP73-1547 | ? | Brazil | BB |
| 239 | SP91-1049 | SP80-3328 | SP81-3250 | Brazil | BB |
| 240 | TUC71-7 | CP52-68 | CP62-258 | Argentina | FH |
| 241 | UBA DEMERARA | *S. sinense* | ? | NA | A |
| 242 | US57-141-5 | *S. robustum* | ? | NA | A |
| 243 | US60-31-3 | 28NG12 | US57-159 | United States | FH |
| 244 | US85-1008 | *S. spontaneum* | US60-31-3 | NA | A |
| 245 | WHITE MAURITIUS | *S. officinarum* | ? | India | A |
| 246 | WHITE PARARIA | *S. barberi* | ? | India | A |
| 247 | WHITE TRANSPARENT | *S. officinarum* | ? | India | A |
| 248 | ZWART MANILA | *S. officinarum* | ? | Indonesia | A |

^1^Accessions number in STRUCTURE analysis (Material and Methods section 2.3). ^2^Information from RIDESA germoplasm bank (http://www.ridesaufscar.com.br), Miami World Collection (http://npgsweb.arsgrin.gov/gringlobal/search.aspx) and Sugarcane Variety Notes (Rossi, 2000). NA: not available information. ^3^Predefined groups: ancestors accessions (A); accessions of *Saccharum* spp. hybrids from Brazilian breeding programs (BB); accessions of *Saccharum* spp. hybrids from foreign breeding programs - foreign hybrids (FH).
