# Supplementary material for: Molecular diversity and genetic structure of Saccharum complex accessions
Source: PLoS One. 2020 May 22;15(5):e0233211. doi: 10.1371/journal.pone.0233211 (PMC7244124; doi:10.1371/journal.pone.0233211)
Supplement: S2 Table — (DOCX) [file pone.0233211.s002.docx]

**S2 Table.** **Names, sequences 5' – 3', genbank ID and the references of the fixed and arbitrary primers that compose TRAP markers.**

| Primers | Names | Sequences (5' – 3') | Genbank ID | References |
| --- | --- | --- | --- | --- |
| Fixed  (forward) | SuSy | GGAGGAGCTGAGTGTTTC | AF263384 | Alwala et al. (2006) |
|  | SuPs | CGACAACTGGATCAACAG | AB001338 |  |
|  | StSy | GGCAAGAAGAAGTTCGAG | AF446084 |  |
|  | COMT | TCGGTCATCATCACCAAGAA | AJ231133.1 | Suman et al. (2012) |
|  | F5H | ACCACCCTACGTGGACTCAG | NM_119790.2 |  |
| Arbitrary  (reverse) | Arbi1-A* | GACTGCGTACGAATTAAT |  | Li and Quiros et al. (2001)  Alwala et al. (2006)  Suman et al. (2012) |
|  | Arbi1-S* | GACTGCGTACGAATTATT |  |  |
|  | Arbi2 | GACTGCGTACGAATTGAC |  |  |
|  | Arbi3 | GACTGCGTACGAATTTGA |  |  |

* Arbi1-A and Arbi1-S were the arbitrary primers Arbi1 used by Alwala et al. (2006) and Suman et al. (2012), respectively.
