# Supplementary material for: Molecular diversity and genetic structure of Saccharum complex accessions
Source: PLoS One. 2020 May 22;15(5):e0233211. doi: 10.1371/journal.pone.0233211 (PMC7244124; doi:10.1371/journal.pone.0233211)
Supplement: S3 Table — (DOCX) [file pone.0233211.s003.docx]

**S3 Table.** **Functional description of the sequences that gave rise to fixed primers of TRAP markers used in this study.**

| **Gene** | **Genbank** | **Homology** | **e-value** | **Pathway** | **Reaction catalysis** |
| --- | --- | --- | --- | --- | --- |
| SuSy | AF263384 | Sucrose synthase 1 (Sobic.010G072300.1) [*S. bicolor*] | 1.40E-156 | Sucrose degradation II | UDP-α-D-glucose + β-D-fructofuranose ↔ sucrose + UDP + H^+^ |
|  |  | Sucrose synthase 2 (Sobic.001G344500.2) [*S. bicolor*] | 7.50E-78 | Sucrose degradation II | UDP-α-D-glucose + β-D-fructofuranose ↔ sucrose + UDP + H^+^ |
|  |  | Sucrose synthase 6 (Sobic.004G357600.1) [*S. bicolor*] | 1.20E-24 | Sucrose degradation II | UDP-α-D-glucose + β-D-fructofuranose ↔ sucrose + UDP + H^+^ |
| SuPS | AB001338 | Sucrose-phosphate synthase (Sobic.004G068400.1) [*S. bicolor*] | 0.0 | Sucrose biosynthesis I  Sucrose biosynthesis II | UDP-α-D-glucose + β-D-fructofuranose 6-phosphate → sucrose 6^F^-phosphate + UDP + H^+^ |
|  |  | Sucrose-phosphate synthase (Sobic.010G205100.1) [*S. bicolor*] | 0.0 | Sucrose biosynthesis I  Sucrose biosynthesis II | UDP-α-D-glucose + β-D-fructofuranose 6-phosphate → sucrose 6^F^-phosphate + UDP + H^+^ |
|  |  | Sucrose-phosphate synthase (Sobic.009G233200.3) [*S. bicolor*] | 2.10E-117 | Sucrose biosynthesis I  Sucrose biosynthesis II | UDP-α-D-glucose + β-D-fructofuranose 6-phosphate → sucrose 6^F^-phosphate + UDP + H^+^ |
|  |  | Sucrose-phosphate synthase (Sobic.003G403300.1) [*S. bicolor*] | 3.0E-33 | Sucrose biosynthesis I  Sucrose biosynthesis II | UDP-α-D-glucose + β-D-fructofuranose 6-phosphate → sucrose 6^F^-phosphate + UDP + H^+^ |
|  |  | Sucrose-phosphate synthase (Sobic.005G089600.1) [*S. bicolor*] | 1.80E-4 | Sucrose biosynthesis I  Sucrose biosynthesis II | UDP-α-D-glucose + β-D-fructofuranose 6-phosphate → sucrose 6^F^-phosphate + UDP + H^+^ |
| StSy | AF446084 | Granule-bound starch synthase 1 (Sobic.010G022600.1) [*S. bicolor*] | 5.10E-85 | Starch biosynthesis | ADP-α-D-glucose_[chloroplast stroma]_ + n (1,4-α-D-glucosyl)_(n)[chloroplast stroma]_ → ADP_[chloroplast stroma]_ + n α-amylose_[chloroplast stroma]_ |
|  |  | Starch synthase IIb-2 (Sobic.004G238600.1) [*S. bicolor*] | 1.10E-17 | Glycogen biosynthesis I  Starch biosynthesis | ADP-α-D-glucose + (1,4-α-D-glucosyl)_(n)_ ↔ ADP + (1,4-α-D-glucosyl)_(n+1)_ |
|  |  | Granule bound starch synthase IIa (Sobic.002G116000.1) [*S. bicolor*] | 1.70E-2 | Starch biosynthesis | ADP-α-D-glucose_[chloroplast stroma]_ + n (1,4-α-D-glucosyl)_(n)[chloroplast stroma]_ → ADP_[chloroplast stroma]_ + n α-amylose_[chloroplast stroma]_ |
| COMT | AJ231133.1 | Caffeic acid 3-O-methyltransferase (Sobic.007G047300.1) [*S. bicolor*] | 0.0 | Suberin monomers biosynthesis  Phenylpropanoid acid biosynthesis  Scopolin and esculin biosynthesis  Phenylpropanoid biosynthesis | trans-caffeate + S-adenosyl-L-methionine → ferulate + S-adenosyl-L-homocysteine + H^+^  5-hydroxy-coniferaldehyde + S-adenosyl-L-methionine → S-adenosyl-L-homocysteine + sinapaldehyde + H^+^ |
|  |  | Caffeate O-methyltransferase (Sobic.001G346800.1) [*S. bicolor*] | 2.90E-124 | Suberin monomers biosynthesis  Phenylpropanoid acid biosynthesis  Scopolin and esculin biosynthesis  Phenylpropanoid biosynthesis | trans-caffeate + S-adenosyl-L-methionine → ferulate + S-adenosyl-L-homocysteine + H^+^  5-hydroxy-coniferaldehyde + S-adenosyl-L-methionine → S-adenosyl-L-homocysteine + sinapaldehyde + H^+^ |
|  |  | Caffeate O-methyltransferase (Sobic.002G325000.2) [*S. bicolor*] | 6.4E-120 | Suberin monomers biosynthesis  Phenylpropanoid acid biosynthesis  Scopolin and esculin biosynthesis  Phenylpropanoid biosynthesis | trans-caffeate + S-adenosyl-L-methionine → ferulate + S-adenosyl-L-homocysteine + H^+^  5-hydroxy-coniferaldehyde + S-adenosyl-L-methionine → S-adenosyl-L-homocysteine + sinapaldehyde + H^+^ |
| CCR | AJ231134.1 | Cinnamoyl-CoA reductase (Sobic.007G141200.1) [*S. bicolor*] | 6.50E-177 | Phenylpropanoid biosynthesis | 4-coumaroyl-CoA + NADPH + H^+^ → 4-coumaraldehyde + coenzyme A + NADP^+^ |
|  |  | Cinnamoyl-CoA reductase (Sobic.010G066000.1) [*S. bicolor*] | 1.40E-109 | Phenylpropanoid biosynthesis | 4-coumaroyl-CoA + NADPH + H^+^ → 4-coumaraldehyde + coenzyme A + NADP^+^ |
|  |  | Cinnamoyl-CoA reductase (Sobic.002G146000.1) [*S. bicolor*] | 8.0E-81 | Phenylpropanoid biosynthesis | 4-coumaroyl-CoA + NADPH + H^+^ → 4-coumaraldehyde + coenzyme A + NADP^+^ |
|  |  | Cinnamoyl-CoA reductase (Sobic.004G065600.1) [*S. bicolor*] | 2.3E-43 | Phenylpropanoid biosynthesis | 4-coumaroyl-CoA + NADPH + H^+^ → 4-coumaraldehyde + coenzyme A + NADP^+^ |
