# Supplementary material for: Molecular diversity and genetic structure of Saccharum complex accessions
Source: PLoS One. 2020 May 22;15(5):e0233211. doi: 10.1371/journal.pone.0233211 (PMC7244124; doi:10.1371/journal.pone.0233211)
Supplement: S4 Table — Total number of fragments, number of polymorphic fragments, percentage of polymorphism, polymorphism information content (PIC) value and discriminatory power (DP) value for each of the eight TRAP markers evaluated in the Brazilian Panel of Sugarcane Genotypes (BPSG). (DOCX) [file pone.0233211.s004.docx]

**S4 Table. TRAP genotyping information.** Total number of fragments, number of polymorphic fragments, percentage of polymorphism, polymorphism information content (PIC) value and discriminatory power (DP) value for each of the eight TRAP markers evaluated in the Brazilian Panel of Sugarcane Genotypes (BPSG).

| TRAP markers | Number of fragments | Polymorphic fragments | Percentage of polymorphism | PIC value | DP value |
| --- | --- | --- | --- | --- | --- |
| SuSy + Arbi1-A | 88 | 87 | 98.86 | 0.97 | 0.99 |
| SuSy + Arbi2 | 84 | 81 | 96.43 | 0.97 | 0.99 |
| StSy + Arbi2 | 75 | 72 | 96.00 | 0.97 | 0.99 |
| StSy + Arbi3 | 87 | 86 | 98.85 | 0.98 | 1.00 |
| SuPS + Arbi2 | 44 | 44 | 100.00 | 0.95 | 0.99 |
| SuPS + Arbi3 | 56 | 53 | 94.64 | 0.95 | 0.96 |
| COMT + Arbi1-S | 81 | 81 | 100.00 | 0.98 | 0.99 |
| F5H + Arbi1-S | 80 | 80 | 100.00 | 0.99 | 0.95 |
| Total | 595 | 584 |  |  |  |
| Average | 74.37 | 73 | 98.10 | 0.97 | 0.98 |
