# Supplementary material for: Molecular diversity and genetic structure of Saccharum complex accessions
Source: PLoS One. 2020 May 22;15(5):e0233211. doi: 10.1371/journal.pone.0233211 (PMC7244124; doi:10.1371/journal.pone.0233211)
Supplement: S5 Table — (DOCX) [file pone.0233211.s005.docx]

**S5 Table. Putative exclusive TRAP fragments observed in the Brazilian Panel of Sugarcane Genotypes (BPSG).**

| Groups^1^ | Species | Number of exclusive fragments per TRAP markers | | | | | | | | Total |
| --- | --- | --- | --- | --- | --- | --- | --- | --- | --- | --- |
|  |  | SuSy+  Arbi1-A | SuSy+  Arbi2 | SuPS+  Arbi2 | SuPS+  Arbi3 | StSy+  Arbi2 | StSy+  Arbi3 | COMT+  Arbi1 | F5H+  Arbi1-S |  |
| A | *Erianthus* spp. | 8 | 18 | 3 | 11 | 8 | 0 | 1 | 0 | 49 |
| A | *S. spontaneum* | 1 | 0 | 1 | 0 | 2 | 4 | 0 | 0 | 8 |
| A | *S. officinarum* | 0 | 0 | 0 | 0 | 0 | 1 | 0 | 0 | 1 |
| A | *S. robustum* | 1 | 0 | 0 | 2 | 3 | 0 | 0 | 0 | 6 |
| A | *S. barberi* | 1 | 0 | 0 | 0 | 0 | 0 | 0 | 0 | 1 |
| BB | *S.* spp. hybrids | 2 | 0 | 0 | 0 | 1 | 0 | 0 | 0 | 3 |
| FH | *S.* spp. hybrids | 0 | 0 | 0 | 0 | 0 | 0 | 0 | 0 | 0 |
| Total |  | 13 | 18 | 4 | 13 | 14 | 5 | 1 | 0 | 68 |

^1^ Predefined groups: ancestors accessions (A); accessions of *Saccharum* spp. hybrids from Brazilian breeding programs (BB); accessions of *Saccharum* spp. hybrids from foreign breeding programs (FH).
