# Supplementary figures and images for: Molecular diversity and genetic structure of Saccharum complex accessions
Source: PLoS One. 2020 May 22;15(5):e0233211. doi: 10.1371/journal.pone.0233211 (PMC7244124; doi:10.1371/journal.pone.0233211)

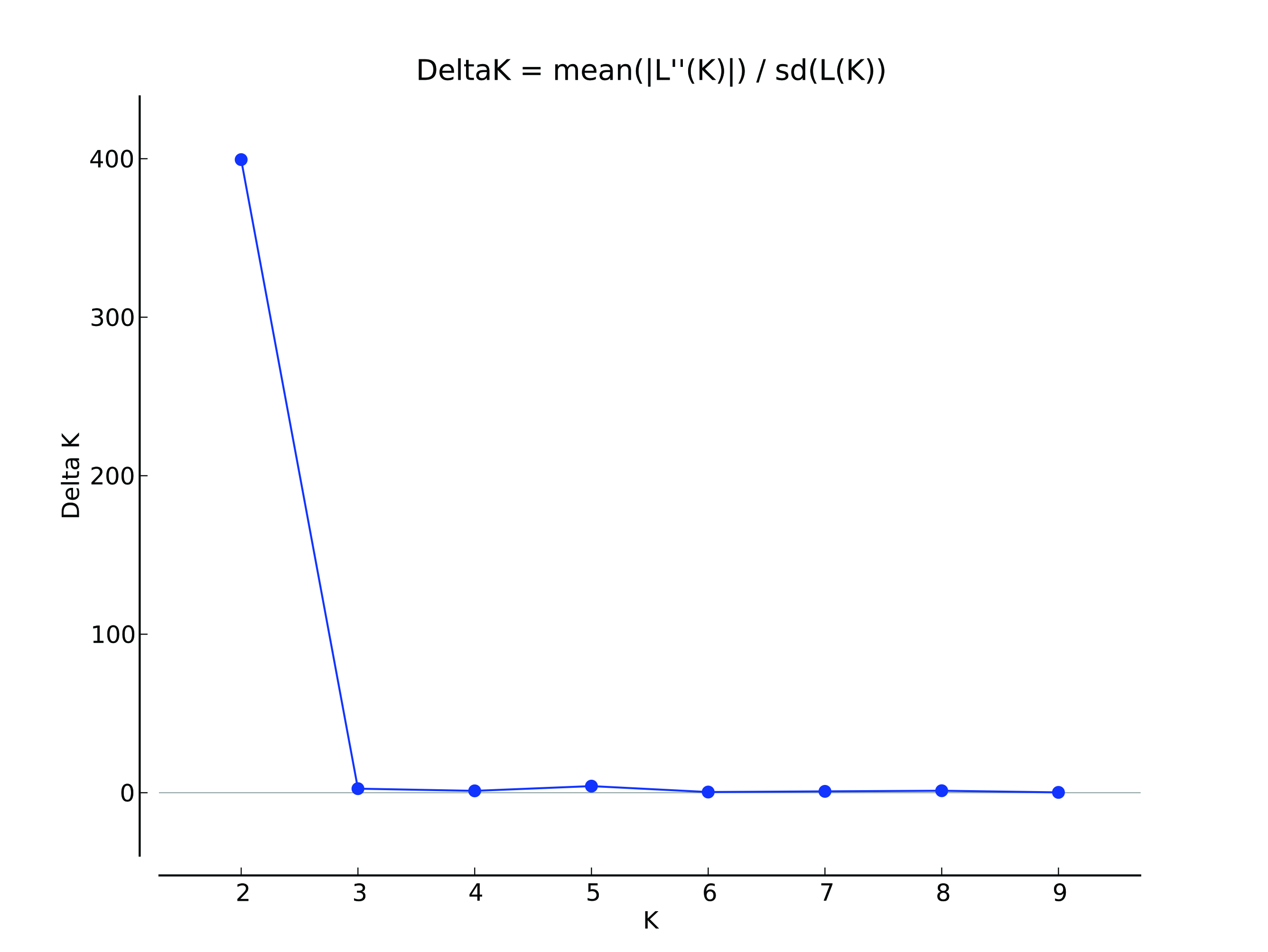

Supplement: S1 Fig — (TIF) [file pone.0233211.s006.tif]

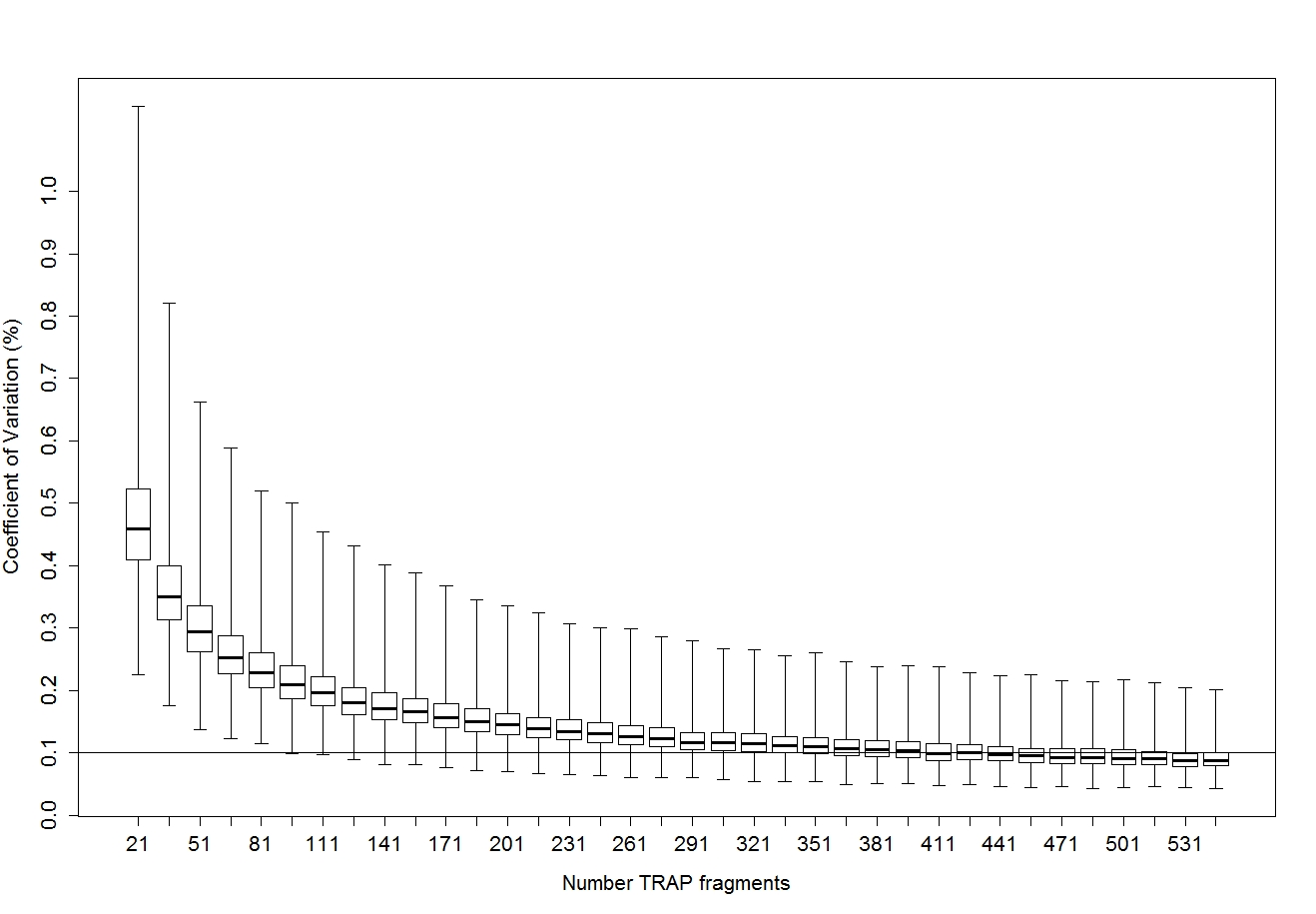

Supplement: S2 Fig — Boxplots of the coefficients of variation (CV%), associated with the estimates of genetic dissimilarities, by bootstrap analysis for subsamples with different numbers of TRAP fragments. (TIF) [file pone.0233211.s007.tif]
