# Supplementary material for: Molecular diversity and genetic structure of Saccharum complex accessions
Source: PLoS One. 2020 May 22;15(5):e0233211. doi: 10.1371/journal.pone.0233211 (PMC7244124; doi:10.1371/journal.pone.0233211)
Supplement: Attachment — Submitted filename: Reviwers comments#.docx [file pone.0233211.s009.docx]

**Reviewer #: Manuscript number: PONE-D-20-08692**

MS title: Molecular diversity and genetic structure of Saccharum complex accessions

**Comments**:

The manuscript submitted to the Journal entitled “*Molecular diversity and genetic structure of Saccharum complex accessions*" is an informative piece of work and seems useful in view of harnessing the potential of this important grass species in breeding and genetics. The work will help the breeders and researcher to raise the information for identification of ideal planting material. The experimentation is properly designed and literature review is up-to-date and reflects the scope of the paper as per scope of Plos one. The conclusions are based on solid data analysis, but still few section in the manuscript is needed revision for clarity and better understanding among the readers. Based on my observations I recommend the manuscripts for minor revision. To improve the manuscript quality, the following comments and suggestions are required to be addressed;

- Bagasse is one of the most industrially useful lignocellulosic byproduct not a ‘’waste’’ (line 15).
- In abstract text is not in sentence case (line 16-17).
- What does it mean by breeding sugarcane history make it clear (line 25) …??
- Abstract is adequately mentioned all about the findings in brief.
- Keywords are not given in pdf version of the MS…?
- Introduction part of the manuscript is nicely documented with relevant citations and reflecting importance of the study.
- Aims of the study are clearly mentioned.
- Plant materials used in the study may be of a great breading value towards the sugarcane genetic improvement.
- How the species is propagated for cultivation. Is it through sexual reproduction or most often it is stem cutting. If though cutting, how genetic material is exchanging genetic information among populations..?
- After adding the ploidy level, the authors should check whether there are any subgroups related with the ploidy level.
- Which type of PCR amplified bands/fragments was treated as putative exclusive TRAP fragments….?
- Table captions are given in bold letters (line 212-213)?
- Result and discussion part is bit lengthy, which can be reduced considerably, Make it brief and crisp.
- Results of Neighbor-Joining-based clustering and phylogenetic analysis, PCA and AMOVA can be correlated, (not statistically but may be observation based). All these parameters are segregating accessions thus, is pattern of clustering/grouping is supporting another...…?
- Authors explained higher dissimilarity value between SES205A (*S. spontaneum*) and CAIANA FITA (*S. officinarum*) accessions (0.62), but as per taxonomic informations *Erianthus* is more distant to genus *Saccharum*…..(310-311)…?
- Discussion part is nicely explained; however it can be brief and crispy.
- Standard format of the references should be followed through-out the MS as per author’s guidelines.
