# Supplementary material for: Molecular diversity and genetic structure of Saccharum complex accessions
Source: PLoS One. 2020 May 22;15(5):e0233211. doi: 10.1371/journal.pone.0233211 (PMC7244124; doi:10.1371/journal.pone.0233211)
Supplement: Attachment — Submitted filename: Response to Reviewers.docx [file pone.0233211.s010.docx]

**Reviewer #: Manuscript number: PONE-D-20-08692**

MS title: Molecular diversity and genetic structure of Saccharum complex accessions

**Comments**:

The manuscript submitted to the Journal entitled “*Molecular diversity and genetic structure of Saccharum complex accessions*" is an informative piece of work and seems useful in view of harnessing the potential of this important grass species in breeding and genetics. The work will help the breeders and researcher to raise the information for identification of ideal planting material. The experimentation is properly designed and literature review is up-to-date and reflects the scope of the paper as per scope of Plos one. The conclusions are based on solid data analysis, but still few section in the manuscript is needed revision for clarity and better understanding among the readers. Based on my observations I recommend the manuscripts for minor revision. To improve the manuscript quality, the following comments and suggestions are required to be addressed;

- Bagasse is one of the most industrially useful lignocellulosic byproduct not a ‘’waste’’ (line 15).

**A:** Revised in the manuscript.

- In abstract text is not in sentence case (line 16-17).

**A:** Revised in the manuscript.

- What does it mean by breeding sugarcane history make it clear (line 25) …??

**A:** In this article, we are focusing on understanding the molecular diversity of the Brazilian Panel of Sugarcane Genotypes A large part of this collection of genotypes originated from Brazilian sugarcane breeding programs. Interestingly, our article showed that it was possible to establish a historical relationship between modern Brazilian varieties of sugarcane (more recent), and their ancestral lines. We want to expose that the PCA analysis suggests clusters that identify the generations of Brazilian breeding accessions (BB), since improved accessions from the first generations of breeding are positioned close to ancestors and improved accessions from the last generations are more distant from the ancestors.

- Abstract is adequately mentioned all about the findings in brief.

**A:** Revised in the manuscript.

- Keywords are not given in pdf version of the MS…?

**A:** Revised in the manuscript.

- How the species is propagated for cultivation. Is it through sexual reproduction or most often it is stem cutting. If though cutting, how genetic material is exchanging genetic information among populations..?

**A:** The exchange of genetic material occurs during the crossing stage between parents. At this stage, genetic recombination occurs and alleles of individuals from different populations can be exchanged. For example, of the cross between RB72454 (Brazilian accession) and TUC71-7 (foreign accession) parents was obtained a the cultivar RB855156. Then, this elite material is propagated vegetatively.

- After adding the ploidy level, the authors should check whether there are any subgroups related with the ploidy level.

**A:** In the present study, there was no addition of the ploidy level, since the TRAP marker was genotyped as dominant (presence or absence of a fragment for a given locus).

- Which type of PCR amplified bands/fragments was treated as putative exclusive TRAP fragments….?

**A:** Fragments that appeared only in a species or breeding group (*Erianthus, S. officinarum, S. spontaneum, S. sinense, S. barberi, S. robustum*, Brazilian breeding programs accessions (BB) and foreign hybrids accessions (FH)) were considered exclusive fragments. In other words, the exclusive fragments were shared by accessions from the same species or breeding group.

- Table captions are given in bold letters (line 212-213)?

**A:** Revised in the manuscript.

- Result and discussion part is bit lengthy, which can be reduced considerably, Make it brief and crisp.

**A:** Revised in the manuscript.

- Results of Neighbor-Joining-based clustering and phylogenetic analysis, PCA and AMOVA can be correlated, (not statistically but may be observation based). All these parameters are segregating accessions thus, is pattern of clustering/grouping is supporting another...…?

**A:** In fact, the different analyses carried out showed a similar tendency for grouping, each with its particularity, but supporting the consistency of the results. The PCA shows in more detail what was pointed out by AMOVA: there is greater genetic differentiation between ancestors accessions (A) and Brazilian breeding programs accessions (BB) than between foreign hybrids accessions (FH) and BB accessions. Therefore, it is natural to see in the PCA (Figure 1B) FH accessions in all quadrants and mostly close to BB accessions. This result is also seen in Neighbor-Joining clustering, since FH accessions are distributed in all clades but also mostly close to BB accessions.

- Authors explained higher dissimilarity value between SES205A (*S. spontaneum*) and CAIANA FITA (*S. officinarum*) accessions (0.62), but as per taxonomic informations *Erianthus* is more distant to genus *Saccharum*…..(310-311)…?

**A:** In the PCA with the six accessions of the *Erianthus* genus (Figure 1A), we found that this genus was responsible for retaining a large part of the data variation and, therefore, it was difficult to see in detail the variation within the genus *Saccharum*, which represents more than 97% of BPSG accessions. In this way, we removed the accessions of *Erianthus* from subsequent analyses, as placed in the section “Materials and methods”, item “Genetic structure” (see below):

“The analysis with STRUCTURE software [47,48], to verify the number of subpopulations ($k$) and the membership proportion ($Q$), was performed considering the 248 accessions of the *Saccharum* genus of BPSG, i.e. without accessions of *Erianthus* genus.”

and

“Finally, the pair-wise dissimilarity among the accessions of the *Saccharum* genus was performed in the R software according to the Jaccard coefficient (Dissimilarity = 1 – Similarity) and a phylogenetic tree was build according to the neighbor-joining (NJ) method with 1,000 bootstrapping through ggtree and ape packages [50, 51].”

and “Discussion” section

“In this study, we removed *Erianthus* accessions from subsequent analysis, since clustering was best viewed when only *Saccharum* accessions were maintained. Despite this, further studies may be conducted to evaluate other regions of the genome closely related to the outstanding traits of the *Erianthus* genus.”

- Discussion part is nicely explained; however it can be brief and crispy.

**A:** Revised in the manuscript.

- Standard format of the references should be followed through-out the MS as per author’s guidelines.

**A:** Revised in the manuscript.
